# Prognostic value of endothelial activation and stress index (EASIX) on short-term and long-term outcomes in type B aortic dissection after thoracic endovascular repair: An observational study

**DOI:** 10.1016/j.xjse.2026.100132

**Published:** 2026-06-18

**Authors:** Xiaosi Chen, Min Liu, Dongqin Cai, Yuan Liu, Nianjin Xie, Songyuan Luo, Jianfang Luo

**Affiliations:** a Department of Cardiology, Guangdong Cardiovascular Institute, Guangdong Provincial People's Hospital, Guangdong Academy of Medical Sciences, Guangdong Provincial Key Laboratory of Coronary Heart Disease Prevention, Guangzhou, P. R. China; b Department of Cardiology, Guangdong Cardiovascular Institute, Guangdong Provincial People's Hospital, Guangdong Academy of Medical Sciences, Southern Medical University, Guangzhou, P. R. China; c Department of Cardiology, School of Medicine, South China University of Technology, Guangzhou, P. R. China

**Keywords:** EASIX, endothelial dysfunction, thoracic endovascular repair, type-B aortic dissection, postoperative complications, all-cause mortality

## Abstract

**Objective:**

The association between endothelial activation and stress index (EASIX) and both short-term and long-term outcomes in patients with type B aortic dissection (TBAD) undergoing thoracic endovascular repair (TEVAR) remains unclear. This study aimed to explore this relationship.

**Methods:**

We conducted a retrospective analysis of 1090 patients with TBAD who underwent TEVAR. Patients were grouped into tertiles according to log2-transformed EASIX. Logistic and Cox regression, win ratio, landmark analysis, propensity score matching, and receiver operating characteristic analyses were performed to evaluate the prognostic and predictive value of EASIX for short-term and long-term outcomes.

**Results:**

A total of 172 (15.8%) patients experienced short-term adverse events, and 206 (18.9%) patients died during a median follow-up of 7.04 years. Logistic regression analyses indicated that greater EASIX levels were associated with an increased risk of composite end points (odds ratio, 1.69; 95% CI, 1.37-2.08; *P* < .001), as well as new-onset dialysis, organ ischemia, and 30-day death. In multivariable Cox regression analyses, EASIX was identified as an independent predictor of all-cause mortality (hazard ratio, 1.43; 95% CI, 1.23-1.68; *P* < .001). The combined models incorporating EASIX demonstrated superior predictive performance for short-term outcomes compared with the baseline models (DeLong test *P* < .05). Time-dependent receiver operating characteristic analysis revealed greater combined model area under the curves than baseline model for all-cause mortality at 1-, 3-, 5-, and 10-year follow-up (likelihood ratio test *P* < .001).

**Conclusions:**

EASIX was associated with both short-term and long-term outcomes in patients with TBAD after TEVAR. Therefore, EASIX may serve as a supplementary indicator for risk stratification before intervention.

**Figure:**
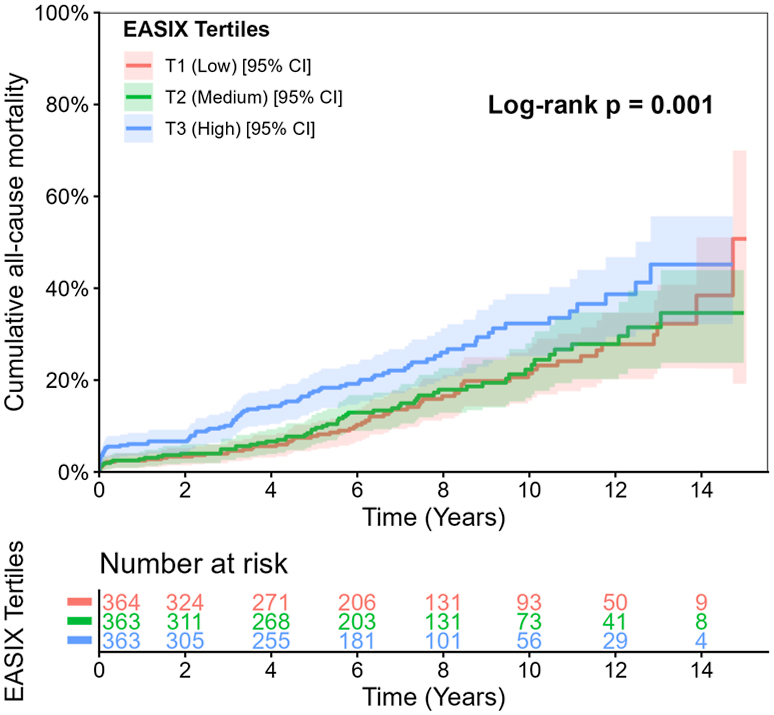
Kaplan-Meier curves for long-term all-cause mortality.

**Central Message**

EASIX is associated with both short-term and long-term outcomes in patients with TBAD after TEVAR. Adding EASIX to traditional prognostic factors can improve predictive performance.

**Perspective**

Endothelial cell dysfunction may be critical in AD prognosis. We found that higher EASIX was associated with increased risks of short-term adverse events and all-cause mortality in patients with TBAD after TEVAR. Adding EASIX to traditional factors can improve predictive performance. Thus, EASIX may be a supplementary indicator for risk stratification before intervention.

See Commentary on page XXX.

Type B aortic dissection (TBAD) is a potentially fatal condition associated with a high prevalence of complications. Current consensus indicates that, under favorable anatomical conditions, thoracic endovascular aortic repair (TEVAR) constitutes a valuable therapeutic strategy for patients with TBAD.^1^ However, the incidence of postoperative mortality and complications remains at a high level during short- and long-term follow-up.^2^ Consequently, early identification of patients at high risk for adverse events after TEVAR, along with optimized perioperative management, is essential for improving clinical outcomes.

Aortic dissection (AD) is characterized by endothelial cell dysfunction, loss of vascular smooth muscle cells, and degradation of elastic fibers in the aortic wall. These alterations can lead to rupture and fatality. The endothelial cell dysfunction and tearing of the intima are critical steps in the development of AD.^3^^,^^4^ Although TEVAR addresses the acute entry tear, it introduces new challenges: stent graft implantation and altered hemodynamics (eg, from residual false lumen) impose sustained mechanical stress and a proinflammatory state that may exacerbate endothelial injury.^5^, ^6^, ^7^ Therefore, preserving endothelial integrity is critical not only for AD pathogenesis but also for recovery after TEVAR. However, endothelial dysfunction (ED) is rarely assessed clinically, underscoring the need for an accessible and objective index for risk stratification in TBAD.

To quantify systemic endothelial impairment, the Endothelial Activation and Stress Index (EASIX)—a noninvasive quantitative biomarker derived from readily available serum measurements of creatinine, lactate dehydrogenase (LDH), and platelet (PLT) count—was initially developed to assess the severity of endotheliopathy in patients undergoing stem cell transplantation.^8^ The EASIX correlation has been elucidated with various endothelial activation markers: chemokine-X-C-ligand 8, interleukin-18, tumorigenicity-2 suppressor, and insulin-like growth factor-1.^8^^,^^9^ Subsequent studies have extended its clinical relevance to systemic conditions such as sepsis^10^ and stroke.^11^ More importantly, accumulating evidence has highlighted its prognostic value in cardiovascular diseases, where ED plays a central role. In this context, EASIX has been associated with adverse outcomes in patients with coronary artery disease (CAD),^12^ acute myocardial infarction,^13^ hypertension,^14^ and aortic stenosis patients undergoing transcatheter aortic valve replacement.^15^ The association between EASIX and the prognosis of patients with TBAD after TEVAR has yet to be explored. To address this gap, this study evaluated the value of EASIX in predicting early and late adverse outcomes.

## Methods

### Patient Population

**Figure 1:**
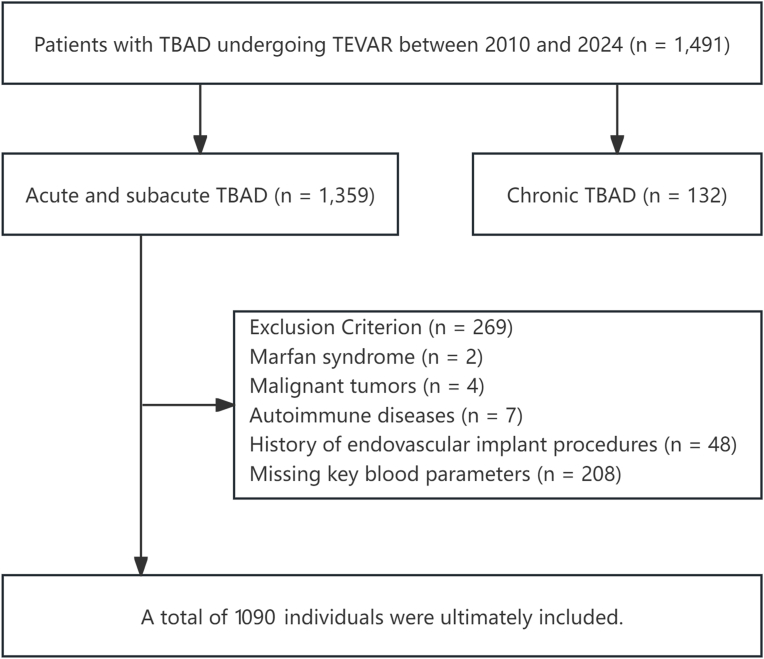
Patient flow chart of study enrollment. History of endovascular implant procedures: percutaneous coronary intervention, coronary artery bypass grafting, TEVAR/EVAR, aortic replacement, or valve replacement. *TBAD*, Type B aortic dissection; *TEVAR*, thoracic endovascular aortic repair; *EVAR*, endovascular aneurysm repair.

This was a single-center study conducted at Guangdong Provincial People's Hospital. Patients with acute and subacute TBAD undergoing TEVAR were consecutively enrolled from January 2010 to January 2024. TBAD was diagnosed using multidetector computed tomography scanning. The exclusion criteria were as follows: (1) chronic TBAD with symptom onset-to-treatment duration >3 months; (2) Marfan syndrome; (3) malignant tumors; (4) autoimmune diseases; (5) history of endovascular procedures; and (6) missing key blood parameters. After we applied these criteria, the final cohort included 1090 patients (Figure 1). The study was approved by the Institutional Ethics Committee of our hospital (date of review: March 29, 2023; approval no. 2019-328H-2), which granted a waiver for informed consent because of the retrospective study design.

### Data Collection and Definitions

Demographics, medical history, comorbidities, laboratory findings, imaging characteristics, and follow-up details were retrospectively recorded and analyzed by 2 researchers. The complete blood count was collected at the time of admission and before TEVAR and measured with an automated blood cell counter (XE-5000; Sysmex). The EASIX parameters, along with the other laboratory values, were obtained from the same blood draw. The EASIX was calculated on the basis of the following formula: LDH (U/L) × creatinine (mg/dL)/PLT (10^9^ cells/L) and log2 conversion for statistical analysis (all EASIX shown in this article are log2 transformed).^8^ According to EASIX tertiles, the participants were divided into 3 distinct groups: T1 (n = 364, EASIX <0.743), T2 (n = 363, 0.743 < EASIX <1.162), and T3 (n = 363, 1.162 < EASIX).

“Acute” TBAD is defined as a condition lasting for <2 weeks, “subacute” as that lasting for 2 weeks to 3 months, and “chronic” as that lasting for >3 months from symptom onset. Complicated TBAD is characterized by dissection with malperfusion of the spinal cord, gastrointestinal tract, the kidneys, or extremities; pleural effusion containing blood, contained or free aortic rupture, persistent pain, and uncontrollable arterial hypertension.^16^ Malperfusion was defined as inadequate blood flow to the end organs as a result of dissection-related obstruction of the aorta and/or its branches.

### TEVAR Indication and Brief Procedure

All patients received optimal medications and underwent TEVAR, as recommended by the guidelines hypertension.^16^ TEVAR was recommended for patients with complicated TBAD. For those with uncomplicated TBAD, TEVAR was considered if any of the following criteria were met: primary entry >10 mm, primary entry at the inner curvature, primary entry located <20 mm to the subclavian artery, false lumen diameter >22 mm, or descending thoracic aortic diameter >40 mm.

TEVAR was performed by a multidisciplinary team comprising interventional cardiologists, cardiothoracic surgeons, anesthetists, and intensivists. For patients with proximal landing zones 1 and 2 or an aberrant right subclavian artery, depending on individualized treatment, a chimney stent or aortic arch bypass was added to TEVAR. The proximal landing zone had to be 2 cm in length, and the device at least 15 cm long. The final decision on TEVAR was a consensus reached with the full informed consent of the patients and family. More procedure details regarding the extent of coverage, oversize rate, type of devices, and endoleak management were provided in the supplementary materials.

### Clinical Outcomes

Follow-up was performed either by scheduled ambulatory visits or telephone interviews. Our clinical focus is divided into short-term and long-term outcomes. Short-term outcomes were assessed using a composite end point of adverse events after TEVAR, defined as 30-day death or any in-hospital complication, including cerebral infarction, spinal cord ischemia (SCI), organ ischemia, limb ischemia, new-onset dialysis, delirium, and reoperation. The composite end point was considered met upon the occurrence of any single event. Individual events were also analyzed as separate outcomes. Long-term outcome refers to all-cause mortality during follow-up, which commenced on the date of the TEVAR procedure and continued until the occurrence of all-cause death or the study cutoff date (February 1, 2025). For a patient developing multiple outcomes during hospitalization, only one outcome was recorded.

### Statistical Analyses

Continuous variables are presented as means ± SDs for normally distributed data or as medians (interquartile ranges [IQRs]) for non-normally distributed data. Categorical variables are reported as frequencies and percentages. Differences in continuous or categorical variables between groups were assessed using χ^2^ tests, one-way analysis of variance, or Kruskal-Wallis tests, as appropriate. Logistic and Cox regression analyses were performed to identify independent predictors of short-term and long-term outcomes. Variables with *P* < .1 in the univariate analysis were included in the multivariate analysis. The *P* values for multiple outcomes (excluding composite end point) in logistic regression analyses were adjusted for multiplicity using the Benjamini-Hochberg correction at a false discovery rate (FDR) of 0.05. Short-term adverse events were evaluated using the win ratio method to account for their non-equivalent clinical severity (see Appendix E1). Kaplan-Meier curves were used to estimate the cumulative mortality rate, and the log-rank test was applied to compare differences among the 3 groups. Landmark analysis evaluated temporal changes in long-term mortality risk profiles.

Receiver operating characteristic (ROC) analysis was conducted to evaluate the predictive value of EASIX for short-term adverse events. The DeLong test was used to compare the area under the curve (AUC) between different models. Time-dependent ROC analysis assessed the predictive value of EASIX for all-cause mortality. Model comparisons were performed using the likelihood ratio test. Using variables with *P* < .05 from the multivariate analysis results, a baseline model was constructed (details of variables included in the baseline model are provided in Table E1). On the basis of baseline model, EASIX was incorporated to form the combined model. Statistical analyses were conducted using R software, version 4.5.0.

### Sensitivity Analysis

To address potential selection bias, 1:1 propensity-score matching was performed using the nearest-neighbor algorithm without replacement. Covariates included in the matching model were age, sex, hypertension, CAD, chronic kidney disease (CKD), maximal aortic diameter (MAD), complications, AD stage, and procedures. Postmatching balance between groups was evaluated using the standardized mean difference, with standardized mean difference <0.20 indicating adequate balance.

Furthermore, to address potential residual confounding, multivariable stratified analyses were performed. Patients were stratified by age, sex, CKD, creatinine levels, complicated TBAD, AD stage, MAD, and procedure. These models were adjusted for the aforementioned covariates (excluding the specific stratification variable being analyzed to prevent collinearity). Finally, interaction tests were conducted to determine whether the prognostic value of the EASIX score was modified by these clinical subgroups.

## Results

### Baseline Characteristics of the Participants

Baseline characteristics according to the EASIX tertiles are presented in Table 1. A total of 1090 patients with TBAD who underwent TEVAR were analyzed. The mean age of the participants was 54 years, with 87.3% male and 12.7% female. The most common comorbid condition was hypertension (82.5%), followed by CAD (13.6%), CKD (8.9%), hyperlipidemia (8.5%), and diabetes (6.1%). The mean MAD was 37.3 mm, and the mean EASIX level was 0.9. Among the patients, 537 (49.3%) had complicated TBAD and 876 (80.4%) were in the acute phase at the time of TEVAR.

**Table 1:** Baseline characteristics of TBAD patients grouped by EASIX tertiles

Variables	Total	T1	T2	T3	*P* value
Age, y	54.0 (46.00-63.0)	54.5 (45.8-62.0)	54.0 (46.0-62.0)	55.0 (47.0-65.0)	.319
Sex					<.001
Male	952 (87.3%)	288 (79.1%)	331 (91.2%)	333 (91.7%)	
Female	138 (12.7%)	76 (20.9%)	32 (8.8%)	30 (8.3%)	
Smoking	522 (47.9%)	169 (46.4%)	189 (52.1%)	164 (45.2%)	.141
Comorbidities					
Hypertension	899 (82.5%)	280 (76.9%)	302 (83.2%)	317 (87.3%)	.001
CAD	148 (13.6%)	43 (11.8%)	49 (13.5%)	56 (15.4%)	.363
Diabetes	66 (6.1%)	22 (6.0%)	25 (6.9%)	19 (5.2%)	.647
Hyperlipidemia	93 (8.5%)	25 (6.9%)	29 (8.0%)	39 (10.7%)	.157
CKD	97 (8.9%)	10 (2.7%)	22 (6.1%)	65 (17.9%)	<.001
Laboratory tests					
WBCs, 10^9^/L	10.4 (8.31-12.8)	9.5 (7.8-11.6)	10.6 (8.2-12.9)	11.5 (8.9-14.0)	<.001
RBCs, 109/L	4.4 (4.00-4.8)	4.3 (4.0-4.7)	4.5 (4.1-4.9)	4.4 (3.9-4.9)	.016
PLT, 10^9^/L	207.0 (165.03-277.0)	287.5 (226.8-360.6)	197.7 (169.5-232.5)	164.0 (138.0-203.4)	<.001
DDI, μg/mL	2430.0 (1042.50-4280.0)	1865.0 (677.0-3120.0)	2200.0 (1035.0-3865.0)	3360.0 (1765.0-9715.0)	<.001
ALT, U/L	21.9 (15.00-35.0)	22.0 (15.0-36.0)	21.0 (14.0-32.0)	22.0 (15.0-40.5)	.040
AST, U/L	21.5 (17.00-30.0)	20.8 (16.0-28.0)	21.0 (17.0-28.0)	24.0 (18.0-38.5)	<.001
ALB, g/L	34.4 (30.71-37.5)	34.2 (30.4-37.0)	35.3 (31.8-38.3)	33.5 (30.1-36.9)	<.001
LDH, U/L	184.0 (153.00-227.8)	156.5 (137.8-186.0)	178.0 (155.0-200.2)	234.0 (194.0-326.0)	<.001
Total cholesterol, mg/dL	4.4 (3.66-5.1)	4.3 (3.7-4.9)	4.4 (3.6-5.1)	4.5 (3.7-5.1)	.280
Triglycerides, mg/dL	1.2 (0.96-1.7)	1.2 (0.9-1.6)	1.2 (0.9-1.7)	1.3 (1.0-1.8)	.039
LDL-C, mg/dL	2.7 (2.16-3.2)	2.8 (2.2-3.2)	2.7 (2.1-3.2)	2.7 (2.1-3.2)	.415
HDL-C, mg/dL	1.0 (0.78-1.1)	0.9 (0.7-1.1)	1.0 (0.8-1.2)	1.0 (0.8-1.2)	<.001
BUN, mg/dL	5.9 (4.54-7.8)	5.0 (3.9-6.3)	5.9 (4.6-7.2)	7.5 (5.6-10.8)	<.001
Creatinine, μmol/L	89.8 (73.62-118.0)	73.5 (62.1-86.0)	89.2 (77.0-104.0)	123.0 (95.9-173.2)	<.001
Complicated TBAD	537 (49.3%)	142 (39.0%)	186 (51.2%)	209 (57.6%)	<.001
Acute TBAD	876 (80.4%)	252 (69.2%)	298 (82.1%)	326 (89.8%)	<.001
MAD, mm	37.3 (34.00-42.2)	37.0 (33.3-42.0)	38.0 (34.2-43.0)	38.0 (34.0-42.8)	.156
EASIX	0.9 (0.65-1.3)	0.6 (0.5-0.7)	0.9 (0.8-1.0)	1.5 (1.3-2.2)	<.001
Operative procedure					.157
TEVAR	693 (63.6%)	249 (68.4%)	218 (60.1%)	226 (62.3%)	
TEVAR with aortic arch bypass	302 (27.7%)	88 (24.2%)	113 (31.1%)	101 (27.8%)	
TEVAR with chimney stent	95 (8.7%)	27 (7.4%)	32 (8.8%)	36 (9.9%)	
Surgery period					.157
Period 1	364 (33.4%)	133 (36.5%)	125 (34.4%)	106 (29.2%)	
Period 2	363 (33.3%)	109 (29.9%)	117 (32.2%)	137 (37.7%)	
Period 3	363 (33.3%)	122 (33.5%)	121 (33.3%)	120 (33.1%)	
Short-term outcomes					
Composite end point	172 (15.8%)	36 (9.9%)	40 (11.0%)	96 (26.4%)	<.001
30-d death	19 (1.7%)	3 (0.8%)	4 (1.1%)	12 (3.3%)	.02
Cerebral infarction	28 (2.6%)	6 (1.6%)	2 (0.6%)	20 (5.5%)	<.001
SCI	19 (1.7%)	4 (1.1%)	4 (1.1%)	11 (3.0%)	.072
Limb ischemia	35 (3.2%)	8 (2.2%)	11 (3.0%)	16 (4.4%)	.233
Organ ischemia	15 (1.4%)	3 (0.8%)	2 (0.6%)	10 (2.8%)	.025
New-onset dialysis	28 (2.6%)	1 (0.3%)	5 (1.4%)	22 (6.1%)	<.001
Delirium	79 (7.2%)	14 (3.8%)	17 (4.7%)	48 (13.2%)	<.001
Reoperation	12 (1.1%)	2 (0.5%)	5 (1.4%)	5 (1.4%)	.446

T1: EASIX's first tertile group; T2: EASIX's second tertile group; T3: EASIX's third tertile group. The cohort was divided into 3 equal time intervals on the basis of surgery date: period 1 (January 11, 2010, to November 14, 2013), period 2 (November 15, 2013, to February 13, 2018), and period 3 (February 14, 2018, to June 1, 2024). *TBAD*, Type B aortic dissection; *EASIX*, endothelial activation and stress index; *CAD*, coronary artery disease; *CKD*, chronic kidney disease; *WBC*, white blood cell; *RBC*, red blood cells; *PLT*, platelet; *DDI*, d-dimer index; *ALT*, alanine aminotransferase; *AST*, aspartate aminotransferase; *ALB*, albumin; *LDH*, lactate dehydrogenase; *LDL-C*, low-density lipoprotein cholesterol; *HDL-C*, high-density lipoprotein cholesterol; *BUN*, blood urea nitrogen; *MAD*, maximal aortic diameter; *TEVAR*, thoracic endovascular repair.

There were significant differences observed in sex, hypertension, CKD, and whether the TBAD was complicated or in the acute phase at the time of TEVAR. However, there were no differences in age, smoking status, CAD, diabetes, hyperlipidemia, or MAD. Individual EASIX components (LDH, creatinine, and platelets) exhibited a distinct gradient distribution across the 3 tertiles (*P* < .001). In addition, patients in the T3 group had greater levels of white blood cells (WBC), D-dimer index, aspartate aminotransferase, and blood urea nitrogen but lower albumin levels (*P* < .05). Regarding lipid profiles, no significant differences were observed among the 3 groups in total cholesterol or low-density lipoprotein cholesterol levels (*P* > .05), though slightly elevated triglycerides (*P* = .039) and greater high-density lipoprotein cholesterol levels (*P* < .001) were noted in the T3 group.

Furthermore, Table 1 details the adverse events across the EASIX tertiles. Among the 1090 patients, 172 (15.8%) experienced the composite end point. Specifically, 19 patients died within 30 days after TEVAR, 28 developed cerebral infarction, 19 had SCI, 35 had limb ischemia, 15 had organ ischemia, 28 required new-onset dialysis, 79 experienced delirium, and 12 underwent reoperation. Greater EASIX levels were significantly associated with increased rates of the composite end point (*P* < .001), 30-day death (*P* = .02), cerebral infarction (*P* < .001), organ ischemia (*P* = .025), new-onset dialysis (*P* < .001), and delirium (*P* < .001). Although the differences in other complications—including SCI, limb ischemia, and reoperation—did not reach statistical significance (all *P* > .05), a numerical increase in their incidence was observed with greater EASIX levels.

### Short-Term Adverse Events

**Figure E1:**
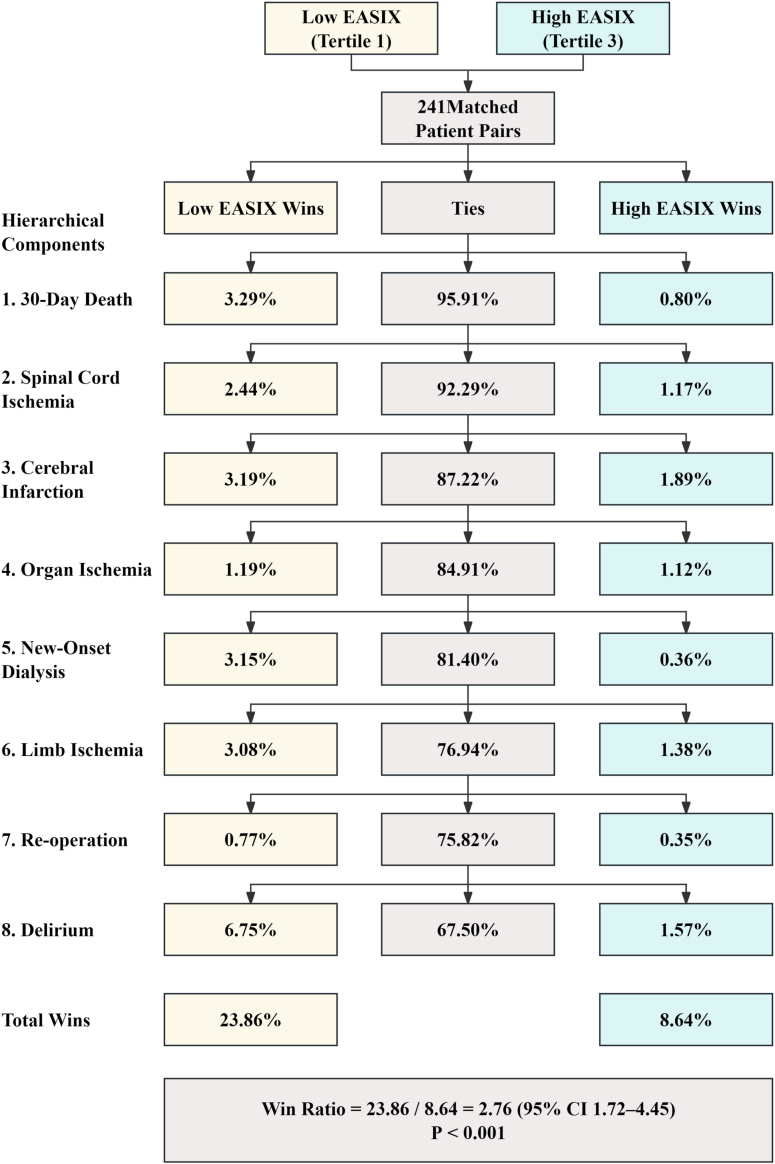
Win ratio diagram for the short-term adverse events. Patients in EASIX tertile 1 were matched 1:1 with those in EASIX tertile 3 using propensity score matching. The matching covariates included sex, hypertension, chronic kidney disease, complicated TBAD, and TBAD stage, which showed baseline imbalances. Within each matched pair, clinical outcomes were evaluated sequentially based on strict clinical severity, prioritizing the most critical events: starting with 30-day death (highest priority) down to delirium (lowest priority). *EASIX*, Endothelial activation and stress index; *TBAD*, type B aortic dissection.

The association between the EASIX and short-term adverse events was evaluated using multivariable logistic regression models (Table 2, the results of the full model covariates are shown in Table E2). After we adjusted for covariates, a greater EASIX score was independently associated with an increased risk of the composite end point (odds ratio [OR], 1.69; 95% CI, 1.37-2.08, *P* < .001). Furthermore, after adjusting for multiple testing, EASIX remained significantly associated with new-onset dialysis (OR, 2.28; 95% CI, 1.64-3.17, FDR-adjusted *P* < .001), organ ischemia (OR, 1.89; 95% CI, 1.31-2.71, FDR-adjusted *P* = .002), and 30-day death (OR, 1.56; 95% CI, 1.08-2.25, FDR-adjusted *P* = .048). Conversely, EASIX was not significantly associated with delirium, SCI, limb ischemia, cerebral infarction, or reoperation (all FDR-adjusted *P* > .05). The win ratio for the short-term adverse events was 2.76 (95% CI, 1.72-4.45, *P* < .001) (Figure E1).

**Table 2:** Multivariate logistic regression analysis of EASIX and short-term adverse events

Outcomes	OR (95% CI)	*P* value	FDR-adjusted *P*	Significant FDR
Composite end point	1.69 (1.37-2.08)	<.001	–	–
New-onset dialysis	2.28 (1.64-3.17)	<.001	<0.001	Yes
Organ ischemia	1.89 (1.31-2.71)	<.001	0.002	Yes
30-d death	1.56 (1.08-2.25)	.018	0.048	Yes
Delirium	1.23 (0.96-1.58)	.095	0.152	No
Spinal cord ischemia	1.38 (0.93-2.03)	.107	0.171	No
Limb ischemia	1.29 (0.92-1.80)	.135	0.181	No
Reoperation	1.22 (0.71-2.11)	.475	0.522	No
Cerebral infarction	1.14 (0.77-1.68)	.522	0.522	No

*EASIX*, Endothelial activation and stress index; *OR*, odds ratio; *FDR*, false discovery rate.

**Figure 2:**
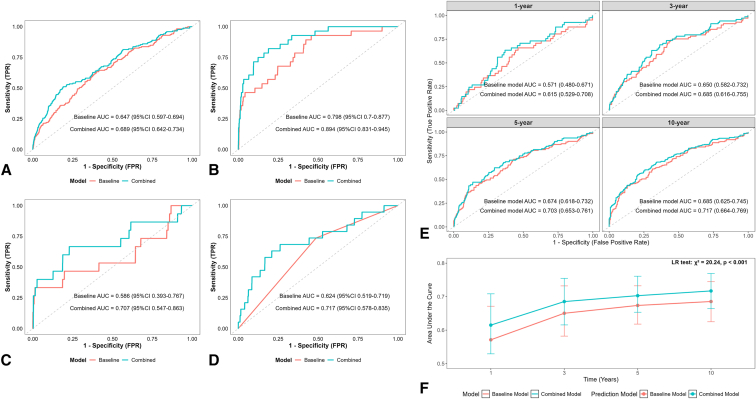
Receiver operating characteristic (*ROC*) analyses of 2 models for predicting short-term adverse events and all-cause mortality. A-D, ROC curves for short-term adverse events, including composite end point (A), new-onset dialysis (B), organ ischemia (C), and 30-day death (D). The baseline model for the composite end point included complicated TBAD, MAD, and WBC; for new-onset dialysis included WBC and RBC; for organ ischemia included DDI and WBCs; and for 30-day death included complicated TBAD. E, Time-dependent ROC curves for predicting all-cause mortality. The baseline model was constructed using age, MAD, WBC, and RBCs. F, Comparison of AUC values between the baseline and combined models at 1-, 3-, 5-, and 10-year follow-up. In all analyses, the combined models were established by adding EASIX to the respective baseline models. *TBAD*, Type B aortic dissection; *MAD*, maximum aortic diameter; *WBC*, white blood cell; *RBC*, red blood cell; *AUC*, area under the curve; *TPR*, true-positive rate; *FPR*, false-positive rate; *LR*, likelihood ratio.

Figure 2, *A-D*, demonstrates the predictive performance of EASIX for early adverse events. ROC analysis was performed to compare the baseline model with a combined model comprising EASIX and baseline predictors. Compared with the baseline model, the combined model demonstrated superior AUC performance across 4 early outcomes. The DeLong test results indicated statistically significant differences between the 2 models for the composite end point (*P* = .008), new-onset dialysis (*P* = .011), and 30-day death (*P* = .025) (Table E3). These findings indicated that incorporating EASIX into traditional predictors significantly enhanced predictive performance. Detailed analyses, including AUC improvements, sensitivity, and specificity, are presented in Table E1.

### Long-Term Mortality

**Figure 3:**
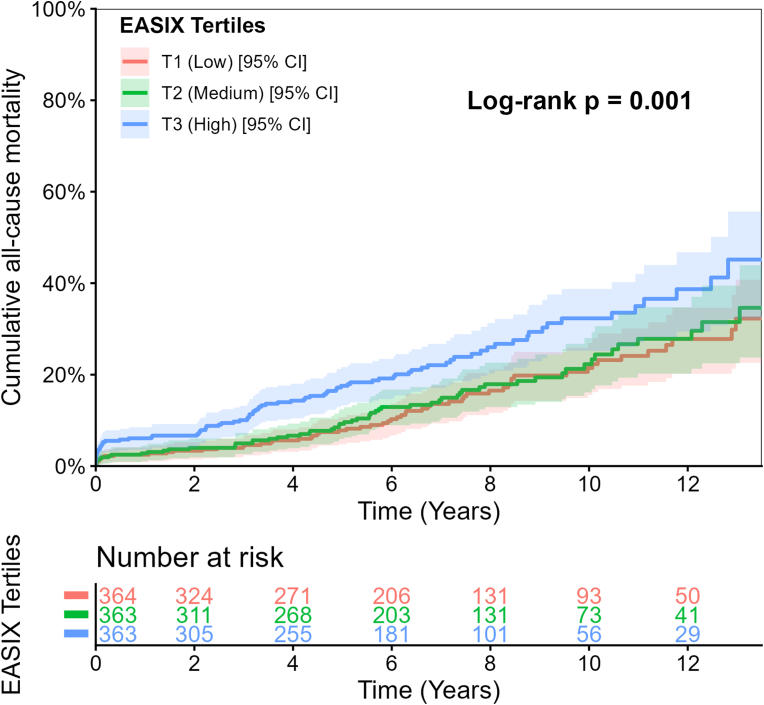
Kaplan-Meier curves for long-term all-cause mortality. T1: EASIX's first tertile group; T2: EASIX's second tertile group; T3: EASIX's third tertile group; The *shaded areas* represent the 95% CIs. *EASIX*, Endothelial activation and stress index.

**Figure E2:**
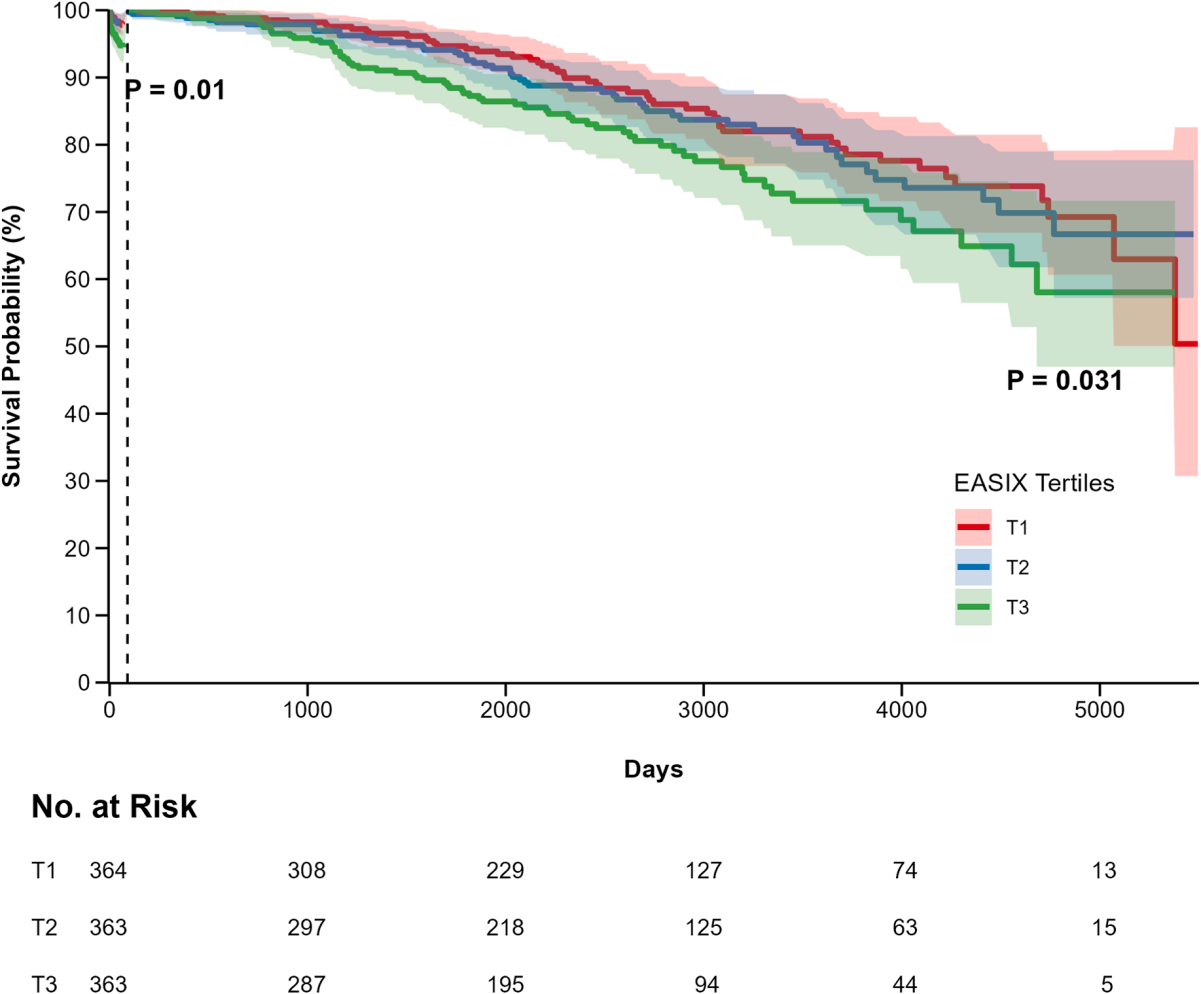
Kaplan-Meier curves for all-cause mortality in the 90-day landmark analysis. Survival time was reset and calculated from day 90. The curves demonstrate that patients in the EASIX tertile 3 continued to experience a significantly greater cumulative incidence of long-term mortality compared to those in the lower tertiles (log-rank *P* = .031). *EASIX*, Endothelial activation and stress index.

During a median follow-up of 7.04 years (IQR, 4.51-10.77), 206 (18.9%) patients experienced all-cause mortality. The Kaplan-Meier survival analysis curves were used to evaluate the incidence of long-term all-cause mortality among various groups on the basis of tertile groupings of EASIX. Compared with the T1 and T2 groups, EASIX in the T3 group has a significantly greater cumulative incidence of all-cause mortality (*P* for log-rank test = .001, Figure 3). The results of the Cox regression analysis for the risk of all-cause death in patients with TBAD are presented in Table 3. EASIX, age, CAD, CKD, MAD, WBC, RBC, albumin, and high-density lipoprotein cholesterol showed significance in univariate analysis (*P* < .05). These variables were included in the multivariate Cox regression analysis. The results showed that EASIX was independently associated with greater all-cause mortality (hazard ratio, 1.43; 95% CI, 1.23-1.68, *P* < .001). In addition, age, MAD, WBC, and RBC were also independently associated with elevated all-cause mortality risk (*P* < .05). Furthermore, a 90-day landmark analysis confirmed that a greater EASIX score remained independently associated with increased risk of all-cause mortality (Table E4, Figure E2).

**Table 3:** Univariate and multivariate Cox regression analysis of EASIX and all-cause mortality

Variables	Univariate predictors		Multivariate predictors	
	HR (95% CI)	*P* value	HR (95% CI)	*P* value
EASIX	1.34 (1.17-1.54)	<.001	1.43 (1.23-1.68)	<.001
Age	1.05 (1.04-1.06)	<.001	1.04 (1.03-1.06)	<.001
CAD	1.87 (1.35-2.58)	<.001	1.23 (0.88-1.73)	.234
CKD	1.71 (1.16-2.52)	.007	1.47 (0.98-2.20)	.066
MAD	1.03 (1.02-1.04)	<.001	1.02 (1.001-1.03)	.041
WBCs	0.93 (0.90-1.97)	.001	0.95 (0.91-0.99)	.015
RBCs	0.58 (0.47-0.71)	<.001	0.78 (0.63-0.97)	.023
ALB	0.95 (0.92-0.97)	<.001	0.99 (0.96-1.02)	.407
HDL-C	0.52 (0.32-0.86)	.010	0.60 (0.35-1.00)	.052

Model fit statistics for the multivariable analysis: C-index = 0.696; Likelihood ratio test *P* < .001. *EASIX*, Endothelial activation and stress index; *HR*, hazard ratio; *CAD*, coronary artery disease; *CKD*, chronic kidney disease; *MAD*, maximal aortic diameter; *WBC*, white blood cell; *RBC*, red blood cell; *ALB*, albumin; *HDL-C*, high-density lipoprotein cholesterol.

Time-dependent ROC analysis was conducted to evaluate the prognostic value of the EASIX for all-cause mortality. The baseline model was constructed using age, MAD, WBC, and RBC, whereas the combined model incorporated EASIX as an additional factor alongside the baseline model. Compared with the baseline model, the combined model demonstrated improved predictive performance at 1 year (0.571 vs 0.615), 3 years (0.650 vs 0.685), 5 years (0.674 vs 0.703), and 10 years (0.685 vs 0.717), as shown in Figure 2, *E*. The likelihood ratio test confirmed that the addition of EASIX significantly improved the model fit (χ^2^ = 20.24, *P* < .001) (Figure 2, *F*). These results suggested that incorporating EASIX into traditional predictive biomarkers appears to enhance predictive value for long-term mortality.

### Sensitivity Analysis

**Figure E3:**
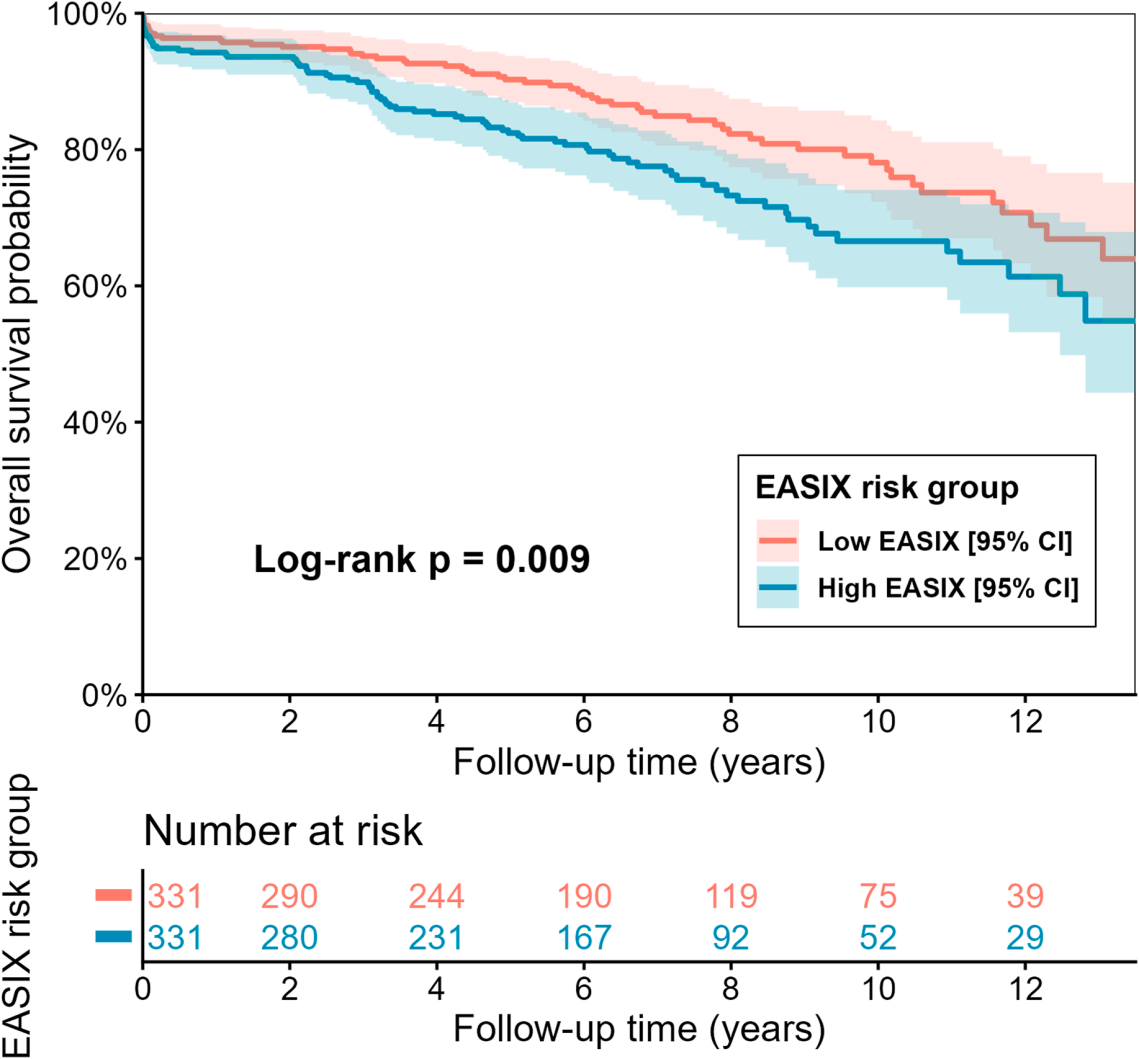
Kaplan-Meier survival curves for all-cause mortality after propensity-score matching. Low EASIX: tertile 1 EASIX and tertile 2 EASIX; High EASIX: tertile 3 EASIX. The *shaded areas* represent the 95% CIs; In the 1:1 propensity score–matched cohort, a total of 662 (331 pairs) patients were included, with 138 (20.1%) deaths from all causes. *EASIX*, Endothelial activation and stress index.

After 1:1 propensity-score matching, 322 patients were included in each group. The baseline characteristics after matching are presented in Table E5. Both Conditional logistic regression and stratified Cox proportional hazards models indicated that the greater-risk EASIX group was significantly associated with a greater risk of short-term and long-term outcomes (Table E6). Compared with the low EASIX group, the high EASIX group had a lower all-cause mortality rate (log-rank *P* = .009, Figure E3).

**Figure 4:**
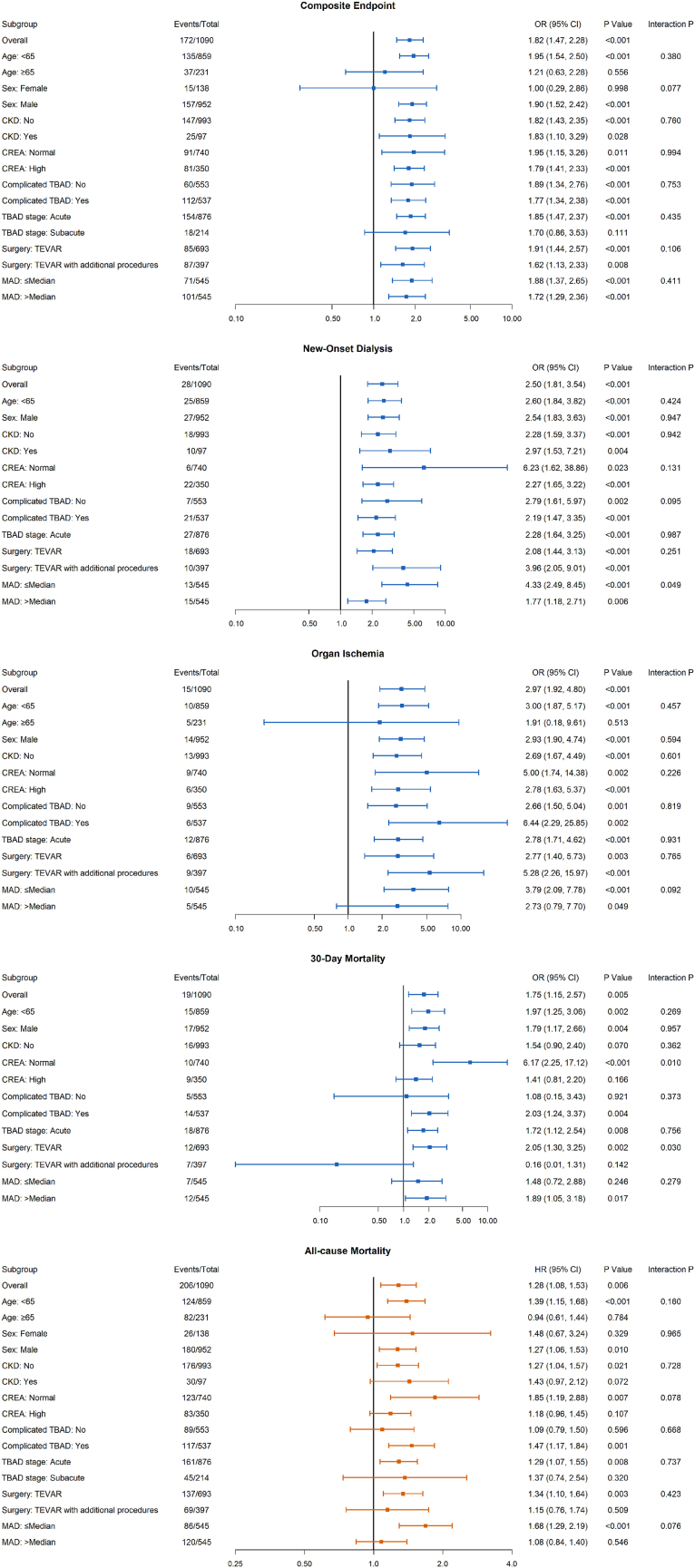
Multivariate stratified analysis of short-term adverse events and all-cause mortality. Short-term adverse events included composite end point, new-onset dialysis, organ ischemia, and 30-day death. Interaction terms were tested using likelihood ratio tests. Stratified variables with fewer than 5 events did not displayed. *OR*, Odds ratio; *CKD*, chronic kidney disease; *CREA*, creatinine; *TBAD*, type B aortic dissection; *TEVAR*, thoracic endovascular aortic repair; *MAD*, maximum aortic diameter; *HR*, hazard ratio.

**Figure E4:**
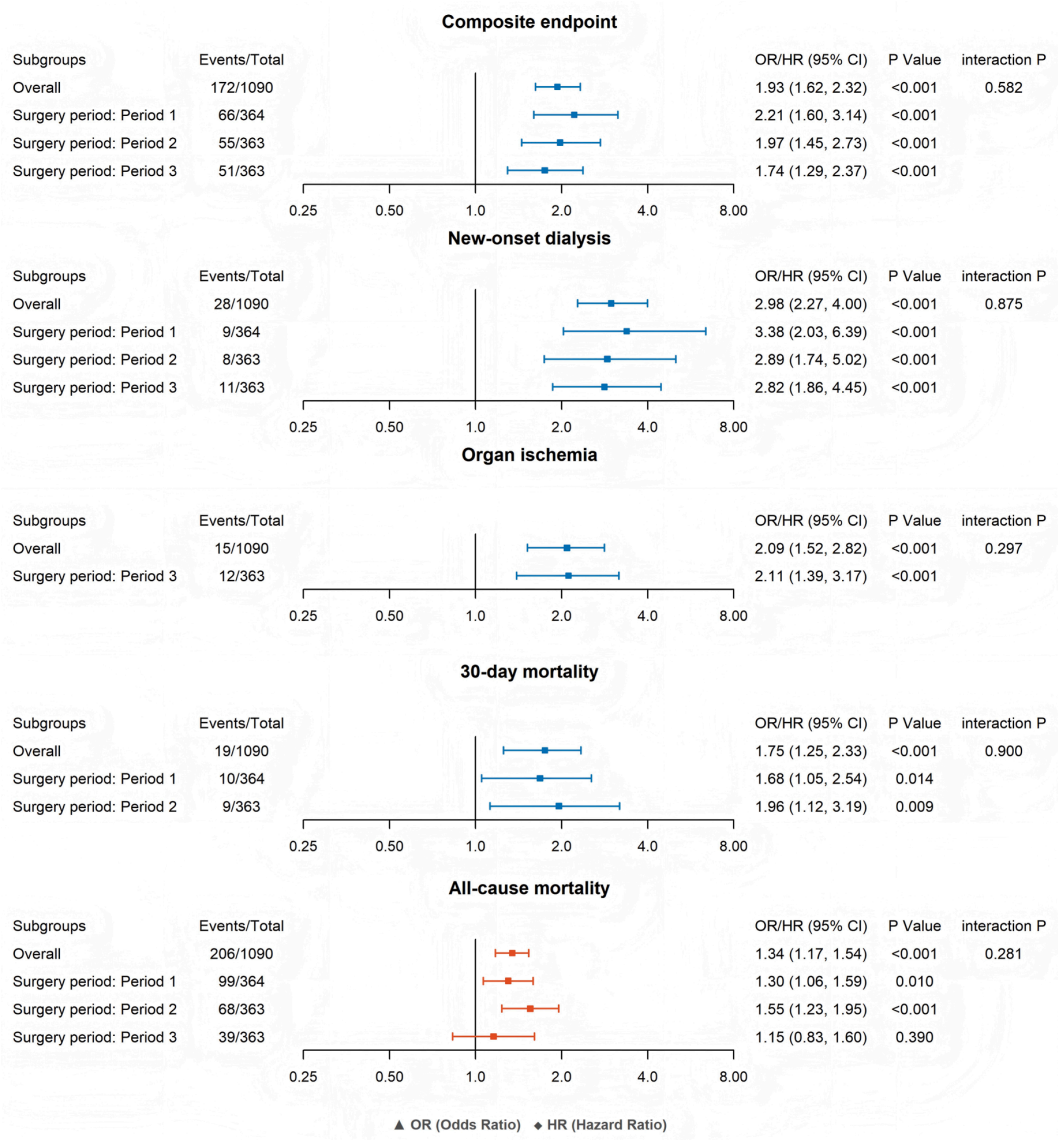
Subgroup analysis for in-hospital complications and all-cause mortality stratified by surgery period. Odds ratios (*ORs*)/hazard ratios (*HRs*) and 95% CIs were calculated per 1-unit increase in EASIX. The cohort was divided into 3 equal time intervals on the basis of surgery date: period 1 (January 11, 2010, to November 14, 2013), period 2 (November 15, 2013, to February 13, 2018), and period 3 (February 14, 2018, to June 1, 2024). Short-term adverse events included composite end point, new-onset dialysis, organ ischemia and 30-day death. Interaction *P* values were calculated using the likelihood ratio test. *EASIX*, Endothelial activation and stress index.

Furthermore, multivariable stratified analyses confirmed the robustness of the EASIX score as an independent prognosticator (Figure 4, Figure E4). After adjusting for potential confounders within each stratum, the high EASIX group was associated with an increased risk of both short-term adverse events (ORs >1.0) and all-cause mortality (hazard ratio >1.0) across the majority of clinical subgroups. Notably, this significant association persisted even in lower-risk subsets, such as patients without CKD, baseline normal creatinine, and without complicated TBAD. In addition, the interaction tests for most subgroups were not statistically significant (*P* for interaction >.05), indicating that the predictive value of the EASIX score was stable.

### Postoperative EASIX Variation and Prognosis Performance

**Figure E5:**
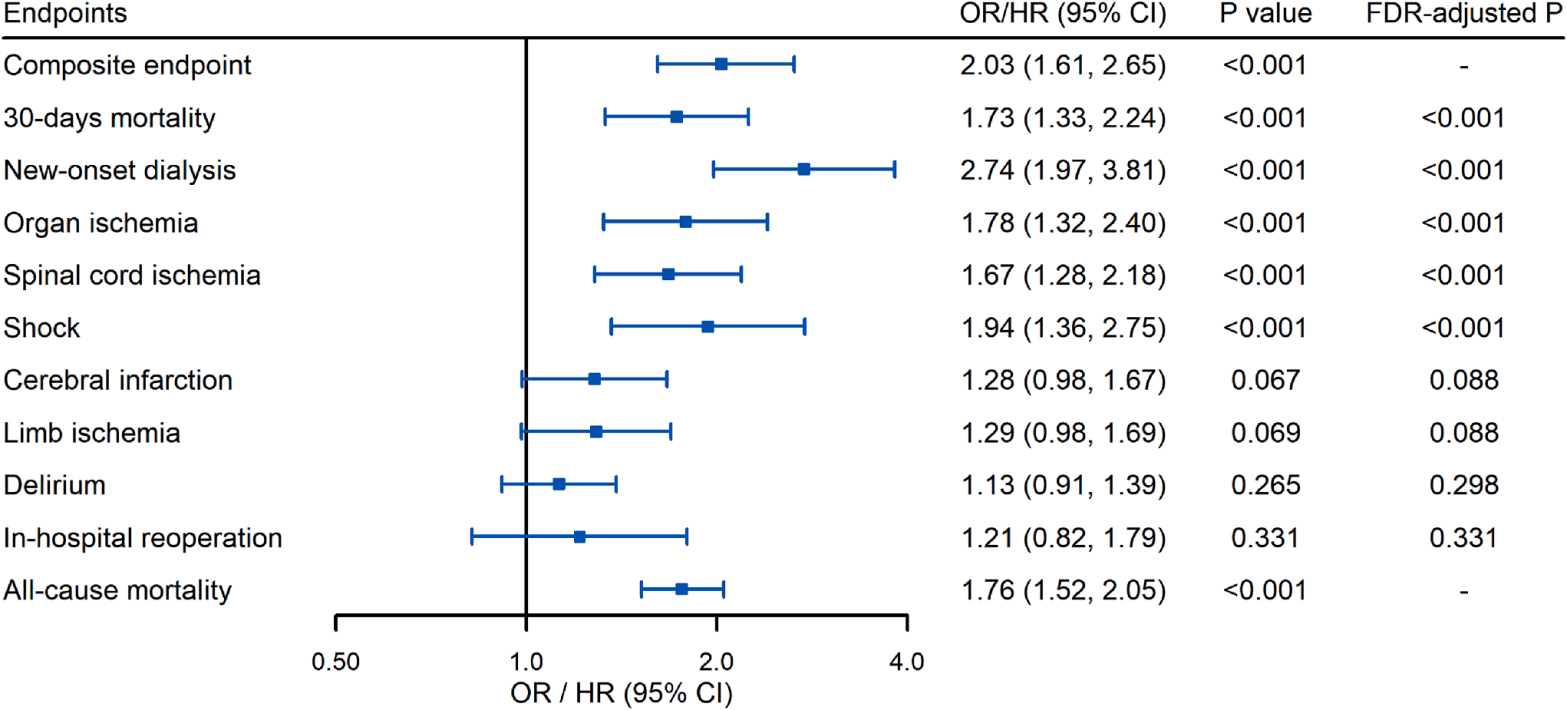
Forest plot of multivariable-adjusted associations between postoperative EASIX and clinical outcomes. The FDR-adjusted *P* values were adjusted for multiplicity using the Benjamini-Hochberg correction at an FDR of 0.05. Multivariate logistic regression analysis was used to evaluate short-term adverse events, whereas the multivariate Cox proportional hazards model was used to assess all-cause mortality. Variables with a *P* value < .1 in the univariate analysis were included in the multivariate analysis. *EASIX*, Endothelial activation and stress index; *OR*, odds ratio; *HR*, hazard ratio; *FDR*, false-discovery rate.

In this retrospective analysis, 587 patients had complete data for postoperative EASIX calculation (Table E7). No significant difference was observed between preoperative and postoperative EASIX levels (*P* = .663). Postoperative EASIX increased significantly across preoperative EASIX tertiles (T1 vs T2 vs T3, *P* < .001). These findings indicate a clear gradient relationship, with higher preoperative EASIX associated with greater postoperative EASIX. Multivariable analyses demonstrated that greater postoperative EASIX was independently associated with the composite end point, 30-day death, new-onset dialysis, organ ischemia, and all-cause mortality (*P* < .001) (Figure E5).

## Discussion

This study identified a significant, independent inverse association between EASIX and short-term and long-term outcomes in patients with TBAD after TEVAR. When EASIX was added to traditional prognostic factors, predictive performance significantly improved. Therefore, EASIX could be considered a useful and relatively simple tool for pre-TEVAR risk stratification and management decisions.

ED is a pivotal driver of aortic wall degradation and intimal tearing. Mechanistically, ED promotes atherosclerosis via inflammatory cell recruitment and matrix degradation^17^ and exacerbates hypertension through impaired nitric oxide bioavailability and increased endothelin-1.^18^ Given that both atherosclerosis and hypertension are well-known risk factors for TBAD, pre-existing ED—as reflected by high EASIX scores—can be used to identify patients at an elevated risk for poor prognosis. Importantly, ED not only triggers the initial TBAD development but also critically limits vascular recovery post-TEVAR. On one hand, anatomical characteristics like a residual false lumen cause incomplete thrombosis and aberrant hemodynamics (eg, oscillatory shear stress), continuously impairing endothelial integrity.^6^^,^^7^ On the other hand, the stent graft itself acts as a foreign body stimulus, independently prolonging vascular inflammation and endothelial activation.^5^ Collectively, these concurrent challenges indicate that ED may persist or even worsen during the post-TEVAR phase, thereby hindering optimal aortic remodeling and driving adverse clinical outcomes.

EASIX, quantified the ED, is a novel biomarker gaining recognition for its prognostic utility. This study is the first to investigate the association between EASIX and TBAD after TEVAR. Our findings indicated that EASIX significantly influenced both short-term and long-term outcomes in patients with TBAD undergoing TEVAR, even after we adjusted for confounding risk factors. The prognostic utility of EASIX likely stems from the synergistic roles of its 3 components: LDH, creatinine, and PLT count. LDH reflects endothelial dysfunction^19^ and serves as a key marker of systemic inflammation.^20^ During inflammation and oxidative stress, LDH is released from endothelial cells, leukocytes, and PLT count, driving adverse vascular remodeling—a critical mechanism in AD progression.^21^^,^^22^ This aligns with evidence linking elevated LDH to increased in-hospital mortality in acute AD.^23^ Concurrently, vascular endothelial cell injury and dysfunction lead to the loss of regulatory function of renal endothelial cells, which have a detrimental impact upon renal function.^24^ One study indicated that impaired renal function, especially as calculated from creatinine levels at discharge, performed as a strong predictor of total mortality among patients with AD.^25^ This is consistent with our findings, in which patients in the greatest EASIX tertile exhibited a greater incidence of new-onset dialysis. Furthermore, reduced PLT levels may result from complement activation and endothelial damage. Endothelial damage exposes subendothelial collagen and elevates tissue factor and von Willebrand factor levels, triggering complement activation and consumptive platelet aggregation.^26^^,^^27^ Previous studies have shown that postoperative thrombocytopenia was an independent predictor of late all-cause mortality for patients with TBAD.^28^ Collectively, the synergistic integration of these 3 parameters comprehensively captures systemic inflammation, renal impairment, and coagulopathy, making EASIX a biologically plausible and reliable prognostic indicator.

The baseline model constructed in this study incorporated statistically significant variables from multivariate analysis (excluding EASIX). Compared with this, superior AUCs were exhibited in the combined model (including EASIX) in both static and time-dependent ROC analyses. Numerous previous studies have identified age,^29^ maximum aortic diameter,^30^ anemia,^31^ WBC, and d-dimer index^32^ as risk factors for poor prognosis in AD. Our findings aligned with the conclusions of these studies, suggesting that EASIX may capture supplementary prognostic information independent of conventional parameters. As a low-cost adjunctive tool, EASIX supplements standard clinical evaluations to refine perioperative risk stratification and monitoring in patients with TEVAR.

Our study highlights EASIX as a rapid and accessible tool for preoperative risk stratification that can be used to guide perioperative management. For patients with high preoperative EASIX scores, hydration to optimize renal clearance, strict blood pressure control, and minimized nephrotoxic exposure can be considered. Postoperatively, reassessment of EASIX components may facilitate early identification of complications such as organ ischemia or dialysis requirement, supporting prolonged intensive care unit or hospital monitoring when necessary. Given its strong association with long-term mortality, patients with a high-EASIX also warrant shorter follow-up intervals and intensive imaging surveillance. Furthermore, endothelial-protective agents—such as statins,^33^ anigotensin-converting enzyme inhibitors,^34^ and sodium-glucose cotransporter-2 inhibitors^35^—can be preferentially administered to patients with TBAD with relevant comorbidities to reduce oxidative stress. Ultimately, incorporating EASIX into routine practice allows for the allocation of medical resources to vulnerable populations.

This study acknowledges several limitations. First, because the study was conducted at a single center in China and only enrolled patients with TBAD who were treated with TEVAR, selection bias and limited generalizability may exist. Second, the retrospective design may have introduced residual confounding factors (eg, residual malperfusion and immediate endoleak classification), despite efforts to adjust for potential confounders. Third, the relationship between EASIX and prognosis is associative rather than causal. Its clinical application warrants cautious interpretation and should be integrated into a comprehensive assessment of therapeutic strategies and patient management. Therefore, future prospective studies involving independent, multicenter cohorts are warranted to establish the robustness and broader clinical applicability of the EASIX. Nevertheless, our institution serves as a high-volume tertiary referral center for aortic diseases, and the large sample size of consecutive TEVAR procedures ensures a representativeness of the study population. In addition, the TEVAR surgical technique and perioperative management strategies were well-established and uniformly applied in our center. Therefore, our data and findings still carry considerable clinical relevance. In addition, the study underscores the clinical value of using routine laboratory parameters for prognostic information. EASIX can serve as an accessible, supplementary indicator for preoperative risk stratification, helping identify patients who are at high risk and guide tailored perioperative management. Furthermore, our study may stimulate investigation into the potential mechanisms underlying the association between EASIX and prognosis, as well as the development of targeted therapies.

## Conclusions

EASIX was associated with both short-term and long-term outcomes in patients with TBAD undergoing TEVAR. Consequently, EASIX may represent a potential supplementary indicator that assists in preoperative risk stratification before intervention.

### Data Availability

Provided there is a valid and reasonable request made for the information derived from this research, it can be obtained from the primary or corresponding author.

## Conflict of Interest Statement

The authors reported no conflicts of interest.

The *Journal* policy requires editors and reviewers to disclose conflicts of interest and to decline handling or reviewing manuscripts for which they may have a conflict of interest. The editors and reviewers of this article have no conflicts of interest.

## Figures and Tables

**Table E1:** The efficacy of EASIX in predicting short-term adverse events

Outcomes	Models	Model variables	AUC (95% CI)	AUC improvement	Sensitivity	Specificity
Composite end point	Baseline model	Complicated TBAD, MAD, WBCs	0.647 (0.601-0.694)	0.043	0.634	0.6
	Combined model	EASIX, Complicated TBAD, MAD, WBCs	0.689 (0.641-0.733)		0.512	0.809
New-onset dialysis	Baseline model	CKD, WBC, RBCs	0.798 (0.713-0.879)	0.096	0.929	0.542
	Combined model	EASIX, CKD, WBC, RBCs	0.896 (0.832-0.946)		0.821	0.811
Organ ischemia	Baseline model	DDI, WBC	0.584 (0.408-0.755)	0.121	0.333	0.986
	Combined model	EASIX, DDI, WBC	0.707 (0.535-0.853)		0.667	0.771
30-d mortality	Baseline model	Complicated TBAD	0.624 (0.517-0.712)	0.093	0.512	0.737
	Combined model	EASIX, Complicated TBAD	0.717 (0.586-0.842)		0.684	0.738

For the 30-day death baseline model, complicated TBAD was retained to ensure the model included at least one reference variable, despite its marginal significance (*P* = .088) in the multivariable analysis. The 95% CIs for the AUCs were estimated using 1000 bootstrap resamples. *EASIX*, Endothelial activation and stress index; *AUC*, area under the curve; *TBAD*, type B aortic dissection; *MAD*, maximum aortic diameter; *WBC*, white blood cell; *RBC*, red blood cell; *DDI*, D-dimer index.

**Table E2:** Results of the full multivariable logistic regression models for short-term adverse events

Outcomes	Covariate	OR	95% CI_lower	95% CI_upper	*P*_value	Model_AIC
Composite end point	Sex	0.68	0.36	1.17	.185	907.97
	Hypertension	1.67	1.00	2.96	.063	907.97
	CKD	1.39	0.82	2.32	.211	907.97
	Complicated TBAD	1.53	1.06	2.22	.024	907.97
	MAD	1.03	1.01	1.05	.002	907.97
	Subacute TBAD	0.61	0.34	1.04	.079	907.97
	WBCs	1.08	1.03	1.13	.001	907.97
	RBCs	0.86	0.66	1.10	.235	907.97
	DDI	1.00	1.00	1.00	.499	907.97
	ALB	0.97	0.94	1.01	.116	907.97
	EASIX	1.69	1.38	2.08	.000	884.937
New onset of dialysis	Hypertension	3.02	0.60	54.90	.287	223.22
	CKD	2.73	1.04	6.65	.032	223.22
	Complicated TBAD	1.36	0.55	3.73	.519	223.22
	Subacute TBAD	0.37	0.02	1.96	.347	223.22
	WBCs	1.19	1.10	1.29	<.001	223.22
	RBCs	0.54	0.29	0.98	.047	223.22
	DDI	1.00	1.00	1.00	.234	223.22
	ALB	0.95	0.87	1.04	.249	223.22
	LDL-C	0.93	0.53	1.58	.786	223.22
	EASIX	2.28	1.64	3.19	.000	201.397
Organ ischemia	WBCs	1.13	1.01	1.24	.018	155.14
	DDI	1.00	1.00	1.00	.050	155.14
	EASIX	1.89	1.28	2.69	.001	147.537
30-d mortality	Complicated TBAD	2.48	0.93	7.84	.088	189.92
	ALB	0.92	0.84	1.01	.093	189.92
	EASIX	1.56	1.05	2.22	.018	186.727

*OR*, Odds ratios; *AIC*, Akaike information criterion; *CKD*, chronic kidney disease; *TBAD*, type B aortic dissection; *MAD*, maximum aortic diameter; *WBC*, white blood cell; *RBC*, red blood cell; *DDI*, D-dimer; *ALB*, albumin; *EASIX*, endothelial activation and stress index; *LDL-C*, low-density lipoprotein cholesterol.

**Table E3:** DeLong test results for receiver operating characteristic models of short-term adverse events

Outcomes	Comparison	AUC1	AUC2	Z statistic	*P* value
Composite end point	Baseline vs combined	0.647	0.689	−2.672534777	.008
New-onset dialysis	Baseline vs combined	0.798	0.894	−2.546089111	.011
Organ ischemia	Baseline vs combined	0.586	0.707	−1.586653109	.113
30-d death	Baseline vs combined	0.624	0.717	−2.242528039	.025

*AUC*, Area under the curve; *AUC1*, baseline model's AUC; *AUC2*, combined model's AUC.

**Table E4:** 90-day landmark analysis of independent predictors for all-cause mortality

Variables	Multivariate predictors	
	HR (95% CI)	*P* value
Age	1.04 (1.03-1.06)	<.001
Coronary artery disease	1.23 (0.87-1.73)	.235
Chronic kidney disease	1.47 (0.98-2.21)	.063
Maximal aortic diameter	1.02 (1.001-1.03)	.037
White blood cells	0.95 (0.91-0.99)	.018
Red blood cells	0.78 (0.63-0.97)	.026
Albumin	0.99 (0.96-1.02)	.410
High-density lipoprotein cholesterol	0.60 (0.36-1.01)	.055
EASIX:strata (time_group) ≤90 d	1.66 (1.30-2.13)	<.001
EASIX:strata (time_group) >90 d	1.35 (1.12-1.63)	.001

The analysis demonstrates that a greater EASIX score remains an independent predictor of all-cause mortality among patients who survive more than 90 days after TEVAR. Hazard ratios (HR) and 95% CIs are presented. *EASIX*, Endothelial activation and stress index.

**Table E5:** Baseline characteristics grouped by EASIX tertiles after propensity score matching

Variables	Total (n = 662)	Low-risk EASIX (n = 331)	High-risk EASIX (n = 331)	*P* value	SMD
Age, y	55.0 (48.00-64.0)	55.0 (48.0-63.0)	56.0 (47.5-65.0)	.535	0.039
Male	611 (92.3%)	305 (92.1%)	306 (92.4%)	1.000	0.011
Smoking	324 (48.9%)	176 (53.2%)	148 (44.7%)	.036	0.170
Comorbidities					
Hypertension	564 (85.2%)	277 (83.7%)	287 (86.7%)	.325	0.085
CAD	95 (14.4%)	43 (13.0%)	52 (15.7%)	.375	0.078
Diabetes	38 (5.7%)	22 (6.6%)	16 (4.8%)	.403	0.078
Hyperlipidemia	55 (8.3%)	21 (6.3%)	34 (10.3%)	.091	0.143
CKD	64 (9.7%)	31 (9.4%)	33 (10.0%)	.895	0.020
Laboratory tests					
WBCs, 10^9^/L	10.8 (8.76-13.2)	10.4 (8.8-12.7)	11.3 (8.9-13.9)	.005	0.267
RBCs, 10^9^/L	4.4 (3.97-4.8)	4.4 (4.0-4.8)	4.4 (3.9-4.9)	.560	0.059
Platelets, 10^9^/L	187.7 (155.00-239.6)	219.0 (180.9-292.0)	161.0 (136.2-195.7)	<.001	0.873
DDI, μg/mL	2660.0 (1190.25-5452.5)	2050.0 (775.0-3580.0)	3190.0 (1735.0-10275.0)	<.001	0.635
ALT, U/L	22.0 (15.00-35.0)	22.0 (15.0-33.8)	22.0 (15.0-38.5)	.332	0.122
AST, U/L	22.2 (17.22-33.0)	21.0 (17.0-29.0)	24.0 (18.0-36.5)	<.001	0.125
ALB, g/L	34.3 (30.52-37.4)	35.0 (31.0-37.8)	33.7 (30.4-37.2)	.021	0.174
LDH, U/L	193.0 (161.00-241.0)	168.0 (146.0-194.0)	233.0 (192.5-315.1)	<.001	0.488
Total cholesterol, mg/dL	4.4 (3.68-5.1)	4.4 (3.7-5.1)	4.4 (3.7-5.0)	.762	0.041
Triglycerides, mg/dL	1.2 (0.96-1.7)	1.2 (0.9-1.6)	1.3 (1.0-1.8)	.059	0.198
LDL-C, mg/dL	2.7 (2.16-3.2)	2.7 (2.2-3.2)	2.7 (2.1-3.2)	.531	0.034
HDL-C, mg/dL	1.0 (0.81-1.2)	1.0 (0.8-1.2)	1.0 (0.8-1.2)	.141	0.095
Creatinine, μmol/L	96.0 (77.00-132.9)	82.0 (68.0-97.0)	120.9 (95.1-163.7)	<.001	0.699
EASIX	1.2 (0.80-1.5)	0.8 (0.6-1.0)	1.5 (1.3-2.2)	<.001	1.640
Disease severity					
Complicated TBAD	378 (57.1%)	191 (57.7%)	187 (56.5%)	.814	0.024
MAD, mm	38.0 (34.00-43.0)	37.6 (34.0-43.0)	38.0 (34.0-43.0)	.950	0.020
Acute TBAD	595 (89.9%)	300 (90.6%)	295 (89.1%)	.606	0.050
Operative procedure					
TEVAR	407 (61.5%)	201 (60.7%)	206 (62.2%)	.749	0.031
TEVAR with additional procedures	255 (38.5%)	130 (39.3%)	125 (37.8%)	.749	0.031
Short outcomes					
Composite end point	127 (19.2%)	42 (12.7%)	85 (25.7%)	<.001	0.335
30-d mortality	14 (2.1%)	5 (1.5%)	9 (2.7%)	.418	0.084
Organ ischemia	12 (1.8%)	4 (1.2%)	8 (2.4%)	.382	0.091
New onset of dialysis	24 (3.6%)	6 (1.8%)	18 (5.4%)	.022	0.195
Delirium	62 (9.4%)	19 (5.7%)	43 (13.0%)	.002	0.251
Cerebral infarction	19 (2.9%)	2 (0.6%)	17 (5.1%)	.001	0.274
Spinal cord ischemia	17 (2.6%)	7 (2.1%)	10 (3.0%)	.623	0.057
Limb ischemia	22 (3.3%)	6 (1.8%)	16 (4.8%)	.051	0.169
Reoperation in-hospital	8 (1.2%)	4 (1.2%)	4 (1.2%)	1.000	<0.001

On the basis of the survival curves observed before propensity-score matching, patients in EASIX tertiles 1 and 2 exhibited similar survival patterns and were therefore combined into a low-risk group, whereas those in tertile 3 formed a high-risk group. *EASIX*, Endothelial activation and stress index; *SMD*, standardized mean difference; *CAD*, coronary artery disease; *CKD*, chronic kidney disease; *WBC*, white blood cell; *RBC*, red blood cell; *DDI*, d-dimer; *ALT*, alanine aminotransferase; *AST*, aspartate aminotransferase; *ALB*, albumin; *LDH*, lactate dehydrogenase; *LDL-C*, low-density lipoprotein cholesterol; *HDL-C*, high-density lipoprotein cholesterol; *MAD*, maximal aortic diameter; *TEVAR*, thoracic endovascular repair; *TBAD*, type B aortic dissection.

**Table E6:** Conditional logistic and stratified Cox regression analyses of adverse outcomes by EASIX groups after propensity score matching

Outcomes	OR/HR (95% CI)	*P* value
Composite end point	2.30 (1.53-3.47)	<.001
New onset of dialysis	3.00 (1.19-7.56)	.020
Organ ischemia	2.00 (0.60-6.64)	.258
30-d mortality	1.80 (0.60-5.37)	.292
Delirium	2.26 (1.32-3.88)	.003
Cerebral infarction	8.50 (1.96-36.79)	.004
Spinal cord ischemia	1.43 (0.54-3.74)	.469
Limb ischemia	2.67 (1.04-6.82)	.041
Reoperation in-hospital	1.00 (0.25-4.00)	1.000
All-cause mortality	1.91 (1.24-2.92)	.003

Odds ratios (ORs), hazard ratios (HR), and 95% CIs were calculated per 1-unit increase in EASIX. *EASIX*, Endothelial activation and stress index.

**Table E7:** Comparison of preoperative and postoperative EASIX levels

Preoperative EASIX (n = 587)	Postoperative EASIX (n = 587)	*P* value
0.99 (0.70-1.41)	0.95 (0.70-1.33)	.663
T1 (n = 172)	0.67 (0.53-0.84)	<.001
T2 (n = 203)	0.95 (0.77-1.17)	
T3 (n = 212)	1.31 (0.93-2.12)	

T1: EASIX's first tertile group; T2: EASIX's second tertile group; T3: EASIX's third tertile group. *EASIX*, Endothelial activation and stress index.

## Appendix E1. Supplementary Methods

### More Procedure Details

#### Extent of coverage

In this research, the proximal anchorage zones placed during thoracic endovascular aortic repair (TEVAR) can be categorized according to the Ishimaru classification. The distribution of proximal coverage segments is as follows: zone 0 (105 cases), zone 1 (220 cases), zone 2 (609 cases), and zone 3 (114 cases). In addition, 26 patients lacked classification information because of incomplete medical record systems or the inability to trace original imaging data. The remaining few patients involved uncommon zones (eg, zone 4 and above). Because of the extremely small sample size, these cases were not listed separately but were included in the overall statistical analysis.

#### Type of devices used

All patients in this cohort received covered stent-grafts as the primary implant for proximal thoracic aortic repair, in accordance with standard TEVAR practice. In selected cases in which distal true lumen collapse or malperfusion was observed intraoperatively, additional bare-metal stents were implanted. This procedure, termed the PETTICOAT technique, dilated the compressed true lumen and improved distal aortic remodeling. The decision to implant a bare metal stent was made on an individual basis according to intraoperative findings and anatomical considerations.

#### Oversize rate

In our center, all TEVAR procedures were performed in accordance with the Chinese clinical guidelines for thoracic endovascular aortic repair. Specifically, the stent graft diameter was generally oversized to the nondissected aortic maximum dimension in the proximal landing zone by 5% to 10% for patients without a chimney stent, but 10% to 15% for patients with a chimney stent, on the basis of preoperative computed tomography angiography measurements.^E1^ Individual variations were permitted on the basis of anatomical characteristics, but no patient received excessive oversizing beyond the guideline recommendations.

#### Immediate endoleak

When postoperative immediate endoleak was observed, it would be managed by balloon dilation, patch stents, coils, or vascular plugs, along with observational follow-up.

### More Details of Statistical Methods

#### Details of the win ratio calculation

To account for the nonequivalent clinical severity of early adverse events after TEVAR, we used the matched-pair win ratio approach.^E2^ First, we performed a 1:1 propensity-score matching to form balanced patient pairs between the high endothelial activation and stress index (EASIX; tertile 3) and low EASIX (tertile 1) groups. We then compared the outcomes within each matched pair. A win was declared for the patient with a better clinical outcome (ie, being free from the adverse event). Each category of the hierarchical early composite end point was evaluated sequentially on the basis of strict clinical severity. In other words, we first considered the occurrence within the pair being compared of (1) 30-day death; if no patient had an event or a tie occurred in category (1), we then sequentially evaluated (2) spinal cord ischemia, (3) cerebral infarction, (4) organ ischemia, (5) new-onset dialysis, (6) limb ischemia, (7) reoperation, and finally (8) delirium. Pairs in which neither patient had a clearly better outcome across all hierarchies were classified as a tie. The win ratio was calculated by dividing the total number of wins in the low EASIX group by the total number of wins in the high EASIX group. A win ratio >1.0 indicates a significantly greater likelihood of experiencing a more favorable early outcome (being free from severe complications) in the low EASIX group compared to the matched high EASIX group.

#### Details of the landmark analysis

To rigorously evaluate whether the longitudinal survival differences were predominantly driven by early procedural mortality, a 90-day landmark analysis was performed. Survival time was reset and calculated from day 90. Kaplan-Meier survival curves were reconstructed, and a landmark multivariable Cox proportional hazards regression model was performed adjusting for the same baseline covariates as the primary analysis, including age, coronary artery disease, chronic kidney disease, maximal aortic diameter, white blood cells, red blood cells, albumin, and high-density lipoprotein cholesterol.

#### Details of the propensity-score matching

For propensity-score matching analysis, conditional logistic regression and stratified Cox regression, with matching pairs as strata, were used to reassess the association between EASIX and short-term and long-term outcomes, respectively.

#### Details of the stratified analyses

For multivariable stratified analyses, the independent associations between EASIX and the short-term and long-term outcomes were reassessed using multivariable logistic regression and Cox proportional hazards models within each stratum, respectively.
